# Association between bariatric surgery and long-term ability to perform household tasks in the Swedish Obese Subjects study: a controlled prospective cohort study

**DOI:** 10.1186/s12916-026-04836-6

**Published:** 2026-04-18

**Authors:** Petra Brembeck, Johanna C. Andersson-Assarsson, Markku Peltonen, Magdalena Taube, Kajsa Sjöholm, Sofie Ahlin, Lucas Adméus, Hanna Konttinen, Ingrid Larsson, My Engström, Björn Henning, Lena M. S. Carlsson, Per-Arne Svensson

**Affiliations:** 1 Institute of Health and Care Sciences, Sahlgrenska Academy at University of Gothenburg, Gothenburg, Sweden; 2 Department of Molecular and Clinical Medicine, Institute of Medicine, Sahlgrenska Academy at University of Gothenburg, Gothenburg, Sweden; 3 Finnish Institute for Health and Welfare, Helsinki, Finland; 4 Department of Clinical Physiology, NU Hospital Group, Region Västra Götaland, Trollhättan, Sweden; 5 Social Psychology, Faculty of Social Sciences, University of Helsinki, Helsinki, Finland; 6 Department of Medicine, Sahlgrenska University Hospital, Region Västra Götaland, Gothenburg, Sweden; 7 Department of Surgery, Sahlgrenska University Hospital, Region Västra Götaland, Gothenburg, Sweden

**Keywords:** Obesity, Bariatric surgery, Home management, Household tasks, Functional status, Long-term outcome

## Abstract

**Background:**

Obesity negatively impacts quality of life and reduces the ability to perform daily activities. While bariatric surgery typically results in sustained weight loss, its long-term impact on functional abilities remains insufficiently studied. The aim of this study was to investigate the long-term association between bariatric surgery and the ability to perform household tasks.

**Methods:**

The ability to perform household tasks was assessed using the standardized Home Management category of the Sickness Impact Profile (SIP) scale in 1641 bariatric surgery patients and 1656 usual obesity care controls from the Swedish Obese Subjects study. Assessments were conducted at baseline and multiple follow-ups over a 20-year period. Patients (aged 37–60 years, BMI ≥ 34 kg/m^2^ for men and ≥ 38 kg/m^2^ for women) were recruited between 1987 and 2001. Analyses were adjusted for sex, and baseline age, BMI, cohabitation status, and weekly working hours as well as year of study inclusion.

**Results:**

At baseline, the score of the Home Management category of the SIP scale was higher in the surgery group than in controls, indicating greater home management dysfunction. Within the first year, however, the surgery group showed significant improvement, attaining a lower score than the control group (4.9 ± 12.4 vs. 7.0 ± 14.1; *p* < 0.001). This improvement persisted throughout 20 years of follow-up, with an adjusted score difference of − 3.2 (95% CI: − 3.9 to − 2.6; *p* < 0.001). Dysfunction in home management followed similar trajectories over time in both sexes but women consistently reported greater dysfunction than men. In the surgery group, individuals who regained weight reported significantly higher dysfunction in home management during 20 years of follow-up than those who maintained weight (adjusted score difference 1.3, 95% CI: 0.2 to 2.4; *p* = 0.020).

**Conclusions:**

Bariatric surgery was associated with improved home management within the first year compared with usual obesity care, and this benefit was sustained over time. These findings suggest that significant sustained weight loss provides lasting improvements in daily functioning.

**Trial registration:**

ClinicalTrials.gov identifier: NCT01479452.

**Supplementary Information:**

The online version contains supplementary material available at 10.1186/s12916-026-04836-6.

## Background

Obesity is associated with a reduced quality of life and numerous physical and psychological health challenges, including limitations in mobility and ability to perform daily activities [1, 2]. Physical functionality is essential for daily living, for example, in completing household tasks, which is a substantial part of daily routines for many people. When the ability to perform such tasks is compromised, it can lead to dissatisfaction and a sense of reduced well-being [2].

Bariatric surgery results in substantial, long-lasting weight loss and is associated with reduced incidences of diabetes [3], cardiovascular disease [4–6], and cancer [7, 8], as well as increased life expectancy [9, 10]. Beyond weight loss and health benefits, studies indicate improvements in quality of life following bariatric surgery [11, 12]. However, certain side effects, such as increased rates of alcohol and substance abuse [13, 14], anemia [15], fractures [16], and weight regain [17], may negatively affect everyday life. While the desire to improve health and enhance various aspects of quality of life often motivates individuals with obesity to pursue weight loss [18–20], the long-term impact of substantial weight loss on everyday functioning, particularly the ability to perform essential household tasks, remains insufficiently studied. The current report addresses this frequently overlooked functional outcome after bariatric surgery: the capacity to manage household tasks. Specifically, we evaluated the ability to perform household tasks using the Home Management category of the Sickness Impact Profile (SIP) scale [21] over a 20-year follow-up period. Our analysis compared individuals with obesity who underwent bariatric surgery to those who received usual obesity care within the prospective, controlled Swedish Obese Subjects (SOS) study.

## Methods

### Study participants and study design

The SOS study is a prospective, non-randomized, matched intervention study comparing long-term outcomes of bariatric surgery and usual obesity care [22]. Between September 1, 1987, and January 31, 2001, a total of 4047 individuals with obesity were included in the study, which was conducted at 25 public surgical departments and 480 primary health-care centers across Sweden. The study protocol was approved by seven ethics review boards in Sweden: Gothenburg, Karolinska Institute, Linköping, Lund, Umeå, Uppsala, and Örebro. All patients provided written or oral informed consent. The study has been registered at ClinicalTrials.gov (NCT01479452). ChatGTP 4.0 Edu was used to improve language to enhance readability.

The control group (*n* = 2037) received usual obesity care provided by the primary health-care centers and the surgery group (*n* = 2010) underwent bariatric surgery. The type of bariatric surgery was decided by the surgeon and consisted of nonadjustable or adjustable gastric banding (*n* = 376), vertical banded gastroplasty (*n* = 1369), or gastric bypass (*n* = 265). The surgery and control groups were matched on the following variables: sex, postmenopausal status, age, smoking status, diabetes, weight, height, hip circumference, waist circumference, systolic blood pressure, triglycerides, total cholesterol, current health, monotony avoidance, psychasthenia, quantity of social support, quality of social support, and stressful life events [22]. The matching was not performed at an individual level. Instead, the matching algorithm selected controls so that the current mean values of the matching variables in the control group became as similar as possible to the current mean values in the surgery group according to the method of sequential treatment assignment [23].

Inclusion criteria were age between 37 and 60 years and a BMI of at least 34 kg/m^2^ for men and 38 kg/m^2^ for women. The exclusion criteria were earlier surgery for gastric or duodenal ulcer, earlier bariatric surgery, gastric ulcer or myocardial infarction during the past 6 months, ongoing malignancy or active malignancy during the past 5 years, bulimic eating pattern, drug or alcohol abuse, psychiatric or cooperative problems contraindicating bariatric surgery, or other contraindicating conditions (for example, anti-inflammatory or continuous glucocorticoid treatments). Patients in the control group who underwent bariatric surgery were censored at the time of this surgery, i.e., data on BMI and Home Management scores after the surgery was not used in the analyses. Similarly, patients in the surgery group who underwent a subsequent procedure to restore normal anatomy were censored at the time of this surgery. Patients without baseline scores for Home Management category of the SIP scale were excluded from the current analysis.

The surgery group was stratified into weight regain and weight maintenance subgroups, based on changes in body weight observed between baseline, the 1-year follow-up, and the 4-year follow-up [17]. Patients who at their 4-year examination had regained 30% or more of the weight they had lost by 1 year were classified into the weight regain group. Those who regained less than 30% of their initial weight loss by year four were assigned to the weight maintenance group. This analysis required body weight data at baseline as well as at the 1- and 4-year follow-ups to allow for assessment of initial weight loss and subsequent weight regain. These time points were selected to represents a balance between allowing sufficient time for weight regain to develop and ensuring adequate follow-up time thereafter. Overall analysis was also performed stratified by sex.

### Outcome variables

The primary endpoint of the SOS study was overall mortality [9, 22]. Data from physical examinations (including body weight measurement), biochemical analyses, and questionnaires (including data on cohabitation status and work time), were measured at baseline and during follow-up at 0.5, 1, 2, 3, 4, 6, 8, 10, 15, and 20 years.

The outcome in the current analysis was dysfunction in ability to perform household tasks, assessed using the Swedish version of the Home Management category of the SIP scale [21, 24, 25]. The overall SIP scale is considered to be a generally good, valid, and reliable instrument to describe functional status, and separate categories, such as the Home Management category, can be used on its own [26]. The Home Management category of the SIP scale is one of 12 SIP categories used to assess how sickness affects a person’s daily life and functioning. It consists of ten weighted items addressing ability to perform household tasks such as cleaning, laundry, gardening, repair work, shopping, sewing, carpentry, and managing household finances [27]. For each item, patients reported whether their ability had deteriorated due to illness. Dysfunction in home management was calculated as a composite score derived from the 10 items in this category. Scores ranged from 0 to 100, with 0 indicating no dysfunction, > 0–10 indicating slight to moderate dysfunction, and > 10–100 indicating marked dysfunction [28]. Thus, higher scores indicated greater health-related dysfunction in home management.

### Statistical analyses

Baseline characteristics are presented as means and standard deviations or as percentages. Changes in BMI and Home Management scores between the groups were analyzed with multilevel mixed-effects regression models. The observations were considered nested within the patient. For individuals who dropped out of the study, we assumed missing data were “missing at random” (i.e., missing data are only dependent on the participant’s observed data), and all the observed data were used in the analyses. Thus, the analyses included all available data at all time points (baseline and follow-up).

Analyses were adjusted for sex, and baseline age, BMI, cohabitation status, and weekly working hours as well as year of study inclusion. Adjustment variables were selected a priori, and no changes were made based on the results of the analyses.

To assess the potential impact of missing data, sensitivity analyses using imputation of missing data were conducted. First, multiple imputation approach was used to predict and impute missing Home Management category data in the whole cohort, using all available Home Management category data at baseline and during the follow-up period, together with sex and baseline age, BMI, cohabitation status, weekly working hours, and year of study inclusion. A total of 30 imputed datasets were generated. Second, the main analyses were repeated with imputation of missing data using worst case scenario, where the missing (censored) data in the control group patients who underwent bariatric surgery during follow-up were assumed to be due to worsening of the home management ability. The missing data for the operated in the control group was thus assumed to be a Home Management category score of 12, indicating markedly worsened dysfunction. For the surgery group, missing data was handled with the multiple imputation approach as described above.

All statistical tests were two-tailed and *p* values of less than 0.05 were considered statistically significant. Stata software, version 18.0 (StataCorp. 2023. Stata Statistical Software: Release 18. College Station, TX: StataCorp LLC.) was used for analyses.

## Results

### Baseline characteristics and BMI changes during follow-up

After excluding individuals with missing baseline scores, 1656 patients remained in the control group and 1641 in the surgery group. Baseline characteristics are summarized in Table 1. Approximately 70% of patients in both groups were women, and about 75% were living with a partner. Compared with the control group, the surgery group was younger, had a higher baseline BMI, a greater prevalence of type 2 diabetes, hypertension, and smoking, and a lower proportion with a university education. Baseline characteristics of the excluded patients are listed in Additional file 1: Table S1.

**Table 1:** Baseline characteristics

	Control group	Surgery group	*p* value
*N*	1656 (50.2%)	1641 (49.8%)	
Age, year (mean, SD)	48.9 (6.3)	47.2 (6.0)	< 0.001
BMI, kg/m^2^ (mean, SD)	40.1 (4.6)	42.4 (4.6)	< 0.001
Home Management category of the SIP scale, score (mean, SD)	8.0 (14.6)	12.0 (16.7)	< 0.001
Working hours/week^a^ (mean, SD)	30.1 (18.1)	28.6 (18.4)	0.018
Sex, women (*n*, %)	1191 (72%)	1178 (72%)	0.938
Living with a partner (*n*, %)	1255 (76%)	1208 (74%)	0.161
University education (*n*, %)	369 (22%)	215 (13%)	< 0.001
Smoking (*n*, %)	317 (19%)	405 (25%)	< 0.001
Diabetes^b^ (*n*, %)	205 (12%)	271 (17%)	< 0.001
Hypertension^c^ (*n*, %)	1017 (61%)	1268 (77%)	< 0.001

^a^Patients with 0 working hours/week included

^b^HbA1c ≥ 48 mmol/mol or fasting blood glucose of ≥ 6.1 mmol/L (≥ 110 mg/dL), or self-reported diabetes medication use

^c^Diastolic blood pressure > 90 mmHg, systolic blood pressure > 140 mmHg, or self-reported antihypertensive medication use

**Fig. 1:**
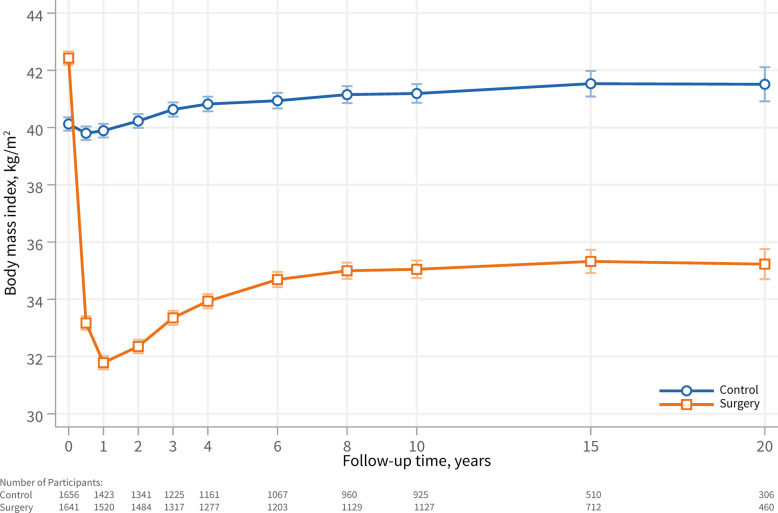
Mean BMI at baseline and during follow-up in the control group and surgery group. Data is presented as adjusted mean BMI and 95% confidence intervals

BMI over 20 years of follow-up is shown in Fig. 1. In the surgery group, BMI decreased drastically within 1–2 years post-surgery. This was followed by partial weight regain, stabilizing at a plateau after 6–8 years. In the control group, BMI changes were small throughout the follow-up period. Number of patients available for analysis at the different time points and major reasons for dropout are listed in Additional file 1: Supplemental Tables S2 and S3.

### Home management dysfunction

**Fig. 2:**
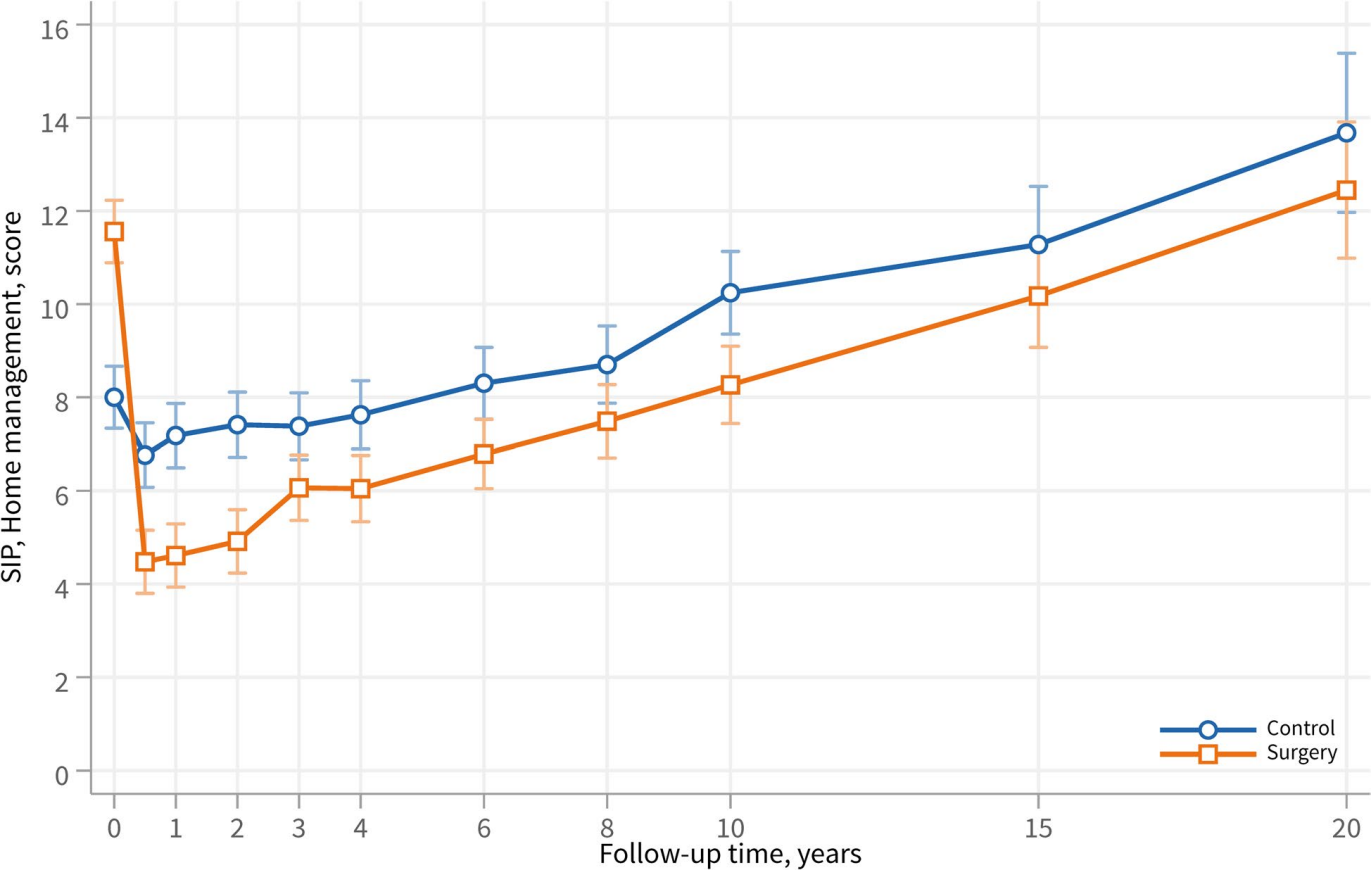
Home management dysfunction over 20 years in the surgery and control groups, as assessed by the Home Management category of the SIP scale. Lower scores indicate a lower level of dysfunction. Data are presented as adjusted mean score and 95% confidence intervals

At baseline, the surgery group reported greater dysfunction in household task performance, as measured by the Home Management category of the SIP scale, compared with the control group (12.0 ± 16.7 vs. 8.0 ± 14.6; *p* < 0.001) (Fig. 2). At the 1-year follow-up, however, the surgery group demonstrated significantly lower dysfunction than the control group (4.9 ± 12.4 vs. 7.0 ± 14.1; *p* < 0.001). From 1 year onwards, both groups showed increasing dysfunction over time; nevertheless, across the entire follow-up period, the surgery group consistently exhibited less dysfunction than the control group (adjusted overall score difference − 3.2, 95% CI − 3.9 to − 2.6; *p* < 0.001).

Results from sensitivity analyses with multiple imputation of missing data showed similar results as the main analyses (multiple imputation: adjusted average difference − 3.1; 95% CI − 3.8 to − 2.3; *p* < 0.001; worst case scenario imputation: − 3.3; 95% CI − 4.1 to − 2.6).

**Fig. 3:**
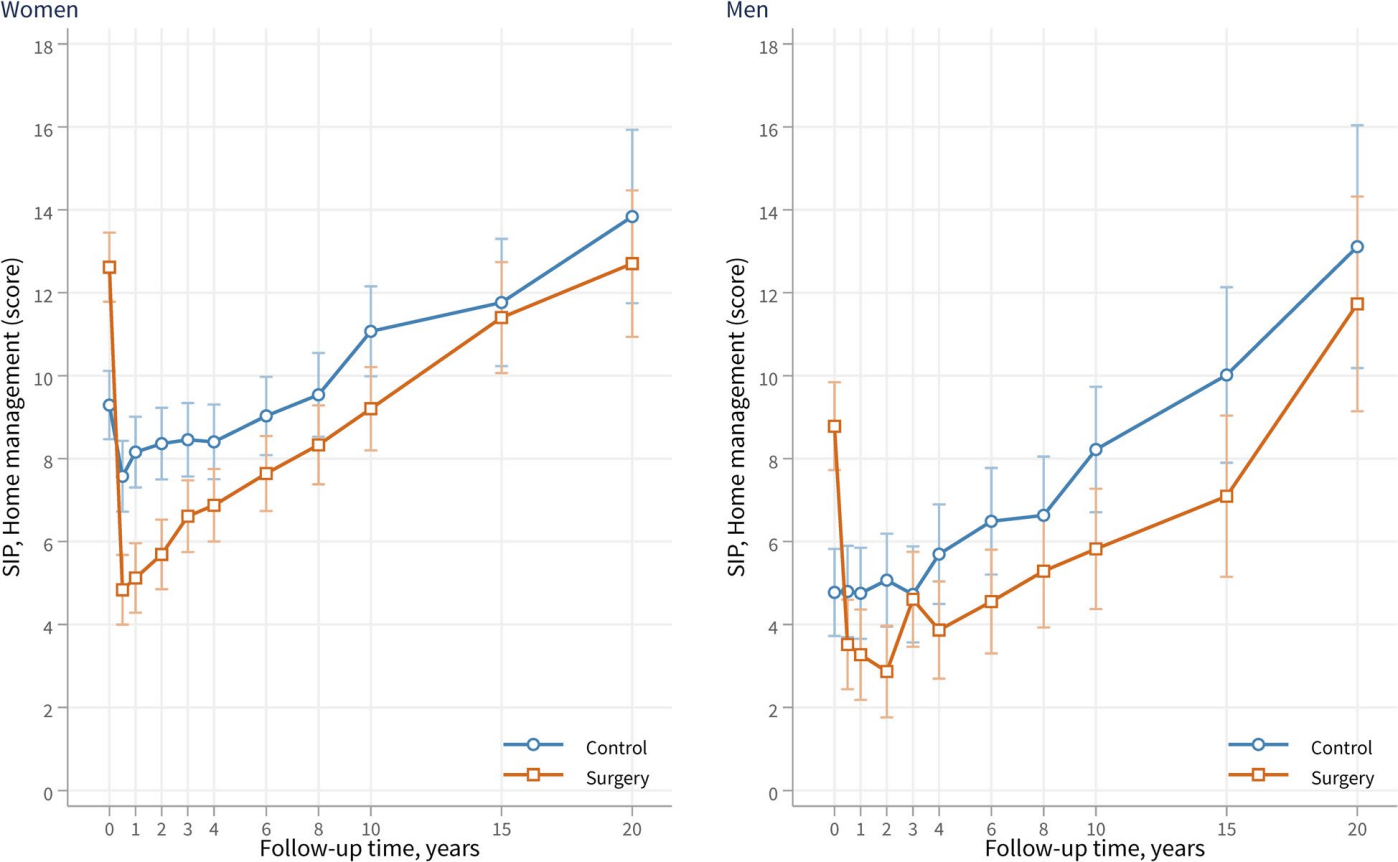
Home management dysfunction over 20 years in women and men of the surgery and control groups, as assessed by the Home Management category of the SIP scale. Lower scores indicate a lower level of dysfunction. Data are presented as adjusted mean score and 95% confidence intervals

Sex-stratified analyses showed that dysfunction in home management was consistently higher in women than in men at all time points in both the surgery and control groups (Fig. 3). However, the score trajectories of the surgery and control groups were comparable across sexes, with the surgery group exhibiting reduced dysfunction. After adjustment, the overall difference between the surgery and control groups during follow-up was − 3.3 (95% CI − 4.1 to − 2.5; *p* < 0.001) for women and − 2.9 (95% CI − 4.0 to − 1.8; *p* < 0.001) for men.

### Home management dysfunction in relation to weight outcomes post-surgery

**Fig. 4:**
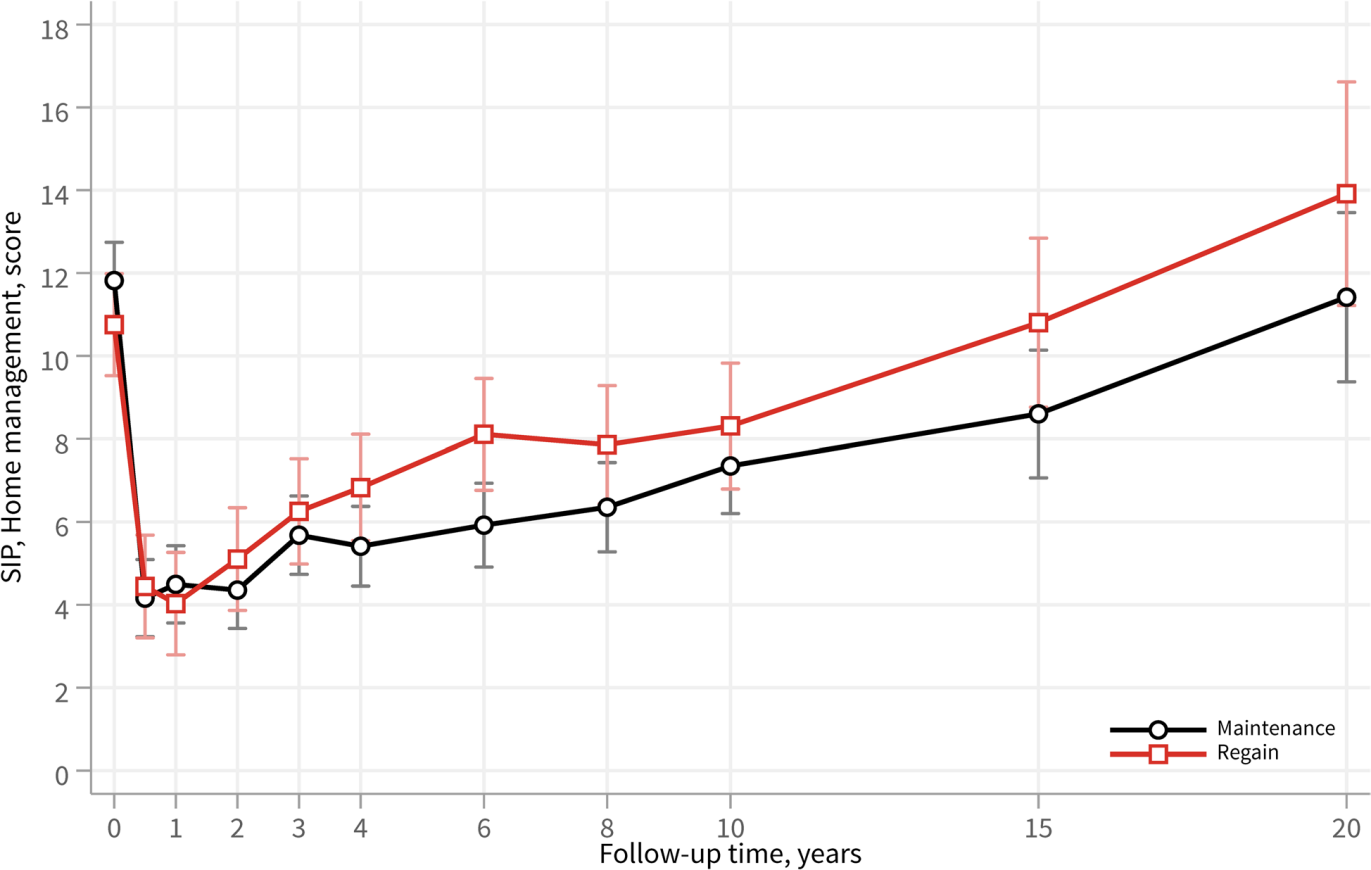
Home management dysfunction over 20 years in the surgery group, stratified by weight regain or weight loss maintenance, as assessed by the Home Management category of the SIP scale. Patients who had regained ≥ 30% of the weight lost at 1 year by the 4-year follow-up were classified as the weight regain group. Lower scores indicate a lower level of dysfunction. Data are presented as adjusted mean scores with 95% confidence intervals

Subgroup analyses were next performed within the surgery group, stratified by weight regain (> 30%) or weight maintenance (< 30%) after surgery (Fig. 4). Patients who regained weight showed significantly greater dysfunction in home management during follow-up compared with those who maintained their weight loss (adjusted mean difference 1.3; 95% CI 0.2–2.4; *p* = 0.020). From the fourth year onward, both groups exhibited a progressive increase in dysfunction over time.

## Discussion

This study demonstrates that bariatric surgery, through substantial and sustained weight loss, is associated with rapid improvements in the ability to perform household tasks among individuals with obesity. These benefits, likely driven by enhanced physical function and overall health, persisted for 20 years compared with usual obesity care. The pattern was consistent across sexes; however, women in both treatment groups consistently reported greater dysfunction than men at all time points. Within the surgery group, patients who regained weight reported greater dysfunction than those who maintained weight loss. Taken together, these findings underscore the long-term positive impact of weight loss and weight maintenance on physical functioning and, potentially, greater independence.

Individuals with obesity are at increased risk for a wide range of conditions and diseases that may negatively affect daily functioning. The Home Management category of the SIP scale is a non–disease-specific instrument widely used to assess changes in health-related functional status across various conditions [21, 27, 29] and is therefore well suited to capture home management dysfunction arising from the diverse comorbidities associated with obesity. For example, it has been used to evaluate home management dysfunction in patients with cancer [27], in whom significant impairment in the Home Management category of the SIP scale has been reported, with a mean score of 15.8 compared with 3.0 in matched controls. In the present study, observed scores were within a comparable range.

At baseline, the surgery group reported greater dysfunction in home management compared with the control group. As the surgery group elected to undergo surgical treatment, this decision may, at least in part, be associated with a higher level of impairment in daily activities at baseline. Within the first postoperative year, the surgery group showed a marked improvement, resulting in scores that were better than those of the control group despite baseline differences. This rapid improvement suggests that weight loss itself likely plays a central role in enhancing capacity for daily activities. The importance of body weight is further supported by our subgroup analyses, which show that patients who regained weight after initial weight loss reported greater dysfunction in home management than those who maintained their weight loss, underscoring the importance of weight status for functional capacity. In the long term, the surgery group consistently exhibited less dysfunction than the control group, possibly reflecting the resolution or prevention of comorbid conditions such as type 2 diabetes and its complications [3, 30], cardiovascular disease [5], and a reduction in work-restricting pain [31] following bariatric surgery. Beyond weight loss and physical health, bariatric surgery often brings broader life changes. Many patients describe the experience as transformative, reporting enhanced self-confidence, increased social interaction and engagement [11, 30, 32], improved self-esteem driven by both physical and psychological changes [33], and even shifts in personal relationships, including cohabitation status [34]. These psychosocial improvements may also contribute to renewed motivation and energy to engage in household tasks.

From 1 year of follow-up onward, both the surgery group and the control group exhibited a gradual increase in dysfunction in home management. This decline likely reflects aging and deteriorating health, consistent with previously documented age-related reductions in physical functioning in the general population [35]. However, we are not aware of other studies examining physical functioning in relation to both age and BMI simultaneously. Despite this trend, the surgery group maintained approximately the same level of dysfunction after 20 years as at baseline, whereas the control group experienced greater dysfunction relative to baseline. To our knowledge, Home Management category of the SIP scale has not previously been evaluated over such an extended follow-up period. Notably, our recent SOS study publication on the Social Interaction category of the SIP scale, based on 15 years of follow-up, found that limitations in social interaction did not follow the same age-related trajectory [11], suggesting that different categories of the SIP scale may be differentially affected by aging.

In both women and men, the surgery and control groups showed similar trends in home management dysfunction over time, although men generally reported lower levels of dysfunction. A Swedish population-based study has also demonstrated sex differences, showing that women, regardless of age, income, employment, or cohabitation, typically devote more time to household tasks such as cooking, cleaning, and laundry [36]. Consequently, limitations in performing these activities may disproportionately affect women, increasing the likelihood of reporting dysfunction in home management. Household responsibilities may also be influenced by factors such as cohabitation status and working hours, and in the present study, results were adjusted for both.

The strengths of the SOS study include its long follow-up with repeated data collection, its large cohort size, and the comparison with a matched control group receiving usual obesity care. The study is limited by relatively high dropout rates at the later follow-up time points, and we cannot rule out residual confounding that may have influenced the results. Another limitation is that societal norms and behaviors related to household tasks have likely changed since the study began, which may reduce the generalizability of the findings to contemporary settings. Nevertheless, because both the surgery and control groups were exposed to the same societal changes, the comparative outcomes remain valid. Another limitation is that the vast majority of patients were of Swedish ancestry, which may restrict the generalizability of the results to more ethnically diverse populations.

## Conclusions

In summary, bariatric surgery was associated with rapid improvements in household task performance within the first year compared with usual obesity care, and these benefits were sustained over time. Household task ability represents a previously underrecognized dimension of the functional benefits of bariatric surgery, underscoring the lasting impact of significant weight loss on daily functioning and overall quality of life.

## Supplementary Information

Additional file 1. Tables S1–S3. Table S1 Baseline characteristics stratified by inclusion into the current analysis. Table S2 Number of participants analyzed at different follow-up time points. Table S3 Cumulative number of persons lost to follow-up over 20 years, stratified by dropout reason.

## Data Availability

The requested information is subject to legal restrictions according to national legislation. Confidentiality regarding personal information in studies is regulated in the Public Access to Information and Secrecy Act (SFS 2009:400), OSL. A request to get access to public documents can be rejected or granted with reservations by the University of Gothenburg. If the University of Gothenburg refuses to disclose the documents the applicant is entitled to get a written decision that can be appealed to the administrative court of appeal.

## Abbreviations

BMI: Body mass index
CI: Confidence interval
SIP: Sickness Impact Profile
SOS: Swedish Obese Subjects
