# Supplementary material for: Association between bariatric surgery and long-term ability to perform household tasks in the Swedish Obese Subjects study: a controlled prospective cohort study
Source: BMC Med. 2026 Apr 18;24:257. doi: 10.1186/s12916-026-04836-6 (PMC13093930; doi:10.1186/s12916-026-04836-6)
Supplement: Supplementary file 1 — Additional file 1. Tables S1–S3. Table S1 Baseline characteristics stratified by inclusion into the current analysis. Table S2 Number of participants analyzed at different follow-up time points. Table S3 Cumulative number of persons lost to follow-up over 20 years, stratified by dropout reason. [file 12916_2026_4836_MOESM1_ESM.pdf]

Additional file 1

Association between bariatric surgery and long-term ability to perform household tasks in the Swedish Obese Subjects study: a controlled prospective cohort study

Brembeck *et al*.

Table S1 Baseline characteristics stratified by inclusion into the current analysis

	Not included	Included	p-value
N	750 (18.5%)	3,297 (81.5%)	
Women	498 (66%)	2369 (72%)	0.003
Age yr	47.5 (5.9)	48.0 (6.2)	0.041
BMI kg/m^2	41.1 (4.8)	41.3 (4.7)	0.491
Inclusion year	1989.4 (1.2)	1995.3 (2.7)	<0.001
Working hours/week	30.8 (18.2)	29.3 (18.1)	0.036
Living with a partner	533 (72%)	2454 (75%)	0.105
Diabetes	131 (17%)	476 (14%)	0.042
Hypertension	587 (78%)	2285 (69%)	<0.001
Current smoking	218 (29%)	722 (22%)	<0.001
University education	104 (14%)	584 (18%)	0.011

Data is presented as number (%) or median (SD)

Table S2. Number of participants analysed at different follow up time points

	Control	Surgery
Baseline	1656	1641
1 years FU	1423 (85.9%)	1520 (92.6%)
2 years FU	1341 (81.0%)	1484 (90.4%)
3 years FU	1225 (74.0%)	1317 (80.3%)
4 years FU	1161 (70.1%)	1277 (77.8%)
6 years FU	1067 (64.4%)	1203 (73.3%)
8 years FU	960 (58.0%)	1129 (68.8%)
10 years FU	925 (55.9%)	1127 (68.7%)
15 years FU	510 (30.8%)	712 (43.4%)
20 years FU	306 (18.5%)	460 (28.0%)

FU= follow up

Table S3 Cumulative number of persons lost to follow-up over 20 years, stratified by dropout reason.

	Control (n=1656)	Surgery (n=1641)
Reoperations	252 (15.2%)	75 (4.6%)
Deaths	309 (18.7%)	260 (15.8%)
Other reasons	789 (47.6%)	846 (51.6%)
